# The evolution of diabetic chronic complications after pancreas transplantation

**DOI:** 10.1186/1758-5996-1-11

**Published:** 2009-09-28

**Authors:** João R de Sá, Patricia T Monteagudo, Érika B Rangel, Cláudio S Melaragno, Adriano M Gonzalez, Marcelo M Linhares, Alcides Salzedas, Maria-Deolinda F Neves, Camila Stela, José O Medina-Pestana

**Affiliations:** 1 Department of Medicine, Division of Endocrinology of Federal University of Sᾶo Paulo, Sᾶo Paulo, Brazil; 2 Department of Medicine, Division of Nephrology of Federal University of Sᾶo Paulo, Sᾶo Paulo, Brazil; 3 Department of Surgery, Division of Gastrosurgery of Federal University of Sᾶo Paulo, Sᾶo Paulo, Brazil

## Abstract

Pancreas transplantation is an invasive procedure that can restore and maintain normoglycemic level very successfully and for a prolonged period in DM1 patients. The procedure elevates the morbimortality rates in the first few months following the surgery if compared to kidney transplants with living donors, but it offers a better quality of life to patients.

Although controversial, several studies have shown the stabilization or the improvement of some of the chronic complications related to diabetes, as well as the extra number of years of life that patients submitted to a double pancreas-kidney transplantation may gain.

Recent studies have demonstrated clashing outcomes regarding isolated pancreas transplantations, a fact which reinforces the need for a more discerning selection of patients for this procedure.

## Introduction

Before 1922, any patient diagnosed with Type 1 Diabetes Mellitus (DM1) had a life expectancy of nearly two more years. However, isolation and industrial-scale insulin production caused a revolution in the treatment of the disease, because it quickly transformed it from fatal to chronic. The increase of life expectancy led to the appearance of chronic complications, which occur from 10 to 20 years after the diagnosis. In the last decades, several medicines, including insulin analogs, have started to be marketed and despite that, DM kept being a disease of difficult control, with most patients presenting A1c targets above the approved levels. Another limiting factor is the higher incidences of hypoglycemia episodes, mainly among patients in intensive insulin therapy. Therefore, despite the improvement of life expectancy in patients under dialysis replacement therapy, DM still persists as the main cause of blindness, non-traumatic amputation of inferior limbs and an important cause of mortality and morbidity secondary to macroangiopathy [1].

One of the possible explanations for the high incidence of complications is the difficulty in obtaining the proper metabolic control for DM1 patients under renal replacement therapy. During a pilot project at our facility, we have noticed that in 76% of the time, patients were off the recommended glycemic target, when evaluated by continuous subcutaneous glucose holter monitoring, performed on three instances: on dialysis day, the previous day and one day after the procedure. Within this context, the pancreas transplantation is an effective alternative, which leads to an improvement in glycemic control, for a prolonged time and with minimal glycemic variation.

Nearly one third of DM patients develop terminal renal failure (TRF) [2] and until mid last century, patients in this condition had few alternatives, once surgeons did not considered them eligible for kidney transplantation due to the lack of glycemic control, which drove, among other complications, the reappearance of renal graft nephropathy. On the other hand, they were considered by general practitioners as patients of difficult metabolic control, due to the progressive loss of the renal function - in other words, little could be done for this subgroup of patients. In 1966, Kelly et cols. [3] performed the first pancreas transplantation in humans. Despite the patient's passing after two months, it was demonstrated that it was possible to reach glycemic control with this procedure. With the improvement of surgical techniques, immunosuppression and multidisciplinary care involved in organ transplantation, the outcomes advanced progressively and, according to current data, over 23,000 pancreas transplantations have been performed worldwide [4]. Brazil currently ranks a spotlight position, with over 800 procedures performed until de end of 2007, and outcomes comparable to most international facilities.

The benefits of pancreas transplantations are the improvement in the quality of life, the prevention of diabetic nephropathy recurrence, the discontinuation of exogenous insulin use and glycemic monitoring, dietary freedom and the potential benefit compared to the evolution of chronic disease-related complications. The major difficulty in discussing the evolution of chronic complications which follow a pancreas transplantation in its several modalities is the lack of randomized clinical trials and a potential bias in patient selection and inclusion, such as the absence of universal transplantation eligibility criteria - mainly, the pancreas transplantation alone, where groups point to bearers of two chronic complications and others to patients with important instability, i.e., potentially more complex. Another parameter that hinders the follow-up is the use of immunosuppressant, which, among other side effects, is potentially nephrotoxic.

The goal of this article is to present data from literature and from our center regarding the evolution of chronic complications that follow pancreas transplantations in its several modalities.

## Diabetic Nephropathy

The risk of microvascular complications caused by diabetes is associated to glycemic control [5] and one of the reasons for the execution of a pancreas transplantation alone (PTA) is the renal protection that results from euglycemia. Fioretto's classic paper written in 1998 [6] demonstrated the importance of the maintenance of normoglycemia in the reversion of histological renal lesions after 10 years in DM1 patients submitted to isolated pancreas transplantation. However, not only is the population of patients studied small, but they have also presented an aggravation of the kidney function, perhaps due to immunosuppression [6] In another study, Farney et cols. [7] followed 97 patients submitted to PTA and it was shown that 9% of them required replacement therapy initiation and were generally patients that, upon entering the study, presented creatinine clearance inferior to 55 ml/minute. The explanations that support the aggravation were associated to immunosuppression, volume contraction due to exocrine bladder drainage, metabolic acidosis, among others.

In 2005, Copelli [8] compared DM1 patients submitted to PTA with DM1 patients kept under intensive insulin therapy (control group), similar in age, gender, diabetes type, and use of statins and anti-hypertensive drugs. Transplanted patients presented better glycemic control, C-peptide level normalization, and improvements in total and LDL cholesterol, and arterial hypertension compared to the control group. Transplanted normoalbuminuric patients remained as such at baseline, and four microalbuminuric patients reverted to normoalbuminuria. Initial clearance was 95 ml/minute and, one year after the procedure, 88 ml/minute - not a significant difference, although indicative of an important improvement in microalbuminuria. This was the first study in literature to demonstrate an improvement in proteinuria, without negatively affecting glomerular filtration rate. Factors related to improvement were glycemic control; BP enhancement and possibly the upswing in C-peptide levels could have resulted in the improvement of endothelial dysfunction due to an increased nitric oxide secretion [8].

These findings are promising, but there is still need for a greater patient sample and an extended follow-up time for the accreditations of the benefits of the procedure. Thus, current medical evidence does not support the indication of PTA for the prevention of diabetic nephropathy, once the nephrotoxicity of immunosuppressants, perhaps linked to volume contraction due to bladder drainage, as well as metabolic acidosis, may cause long-term damage to the renal function, which seems more frequent as the baseline renal function is worse [9].

## Summary: Transplantation and Diabetic Nephropathy

• Normoglycemia for over five years is capable of reverting histological lesions related to DN.

• Renal function may worsen even in patients with normofunctioning pancreas, due to:

- Immunosuppression

- Volume contraction

- Metabolic acidosis

- Hyperglycemic memory

## Diabetic Retinopathy following Pancreas Transplantation

In general, papers show a stabilization of retinopathy in diabetic patients submitted to several modalities of pancreas transplantation. The greatest doubt revolves around proliferative retinopathy (PR), considering that even with reaching glycemic control, nearly 15% of patients presented a worsening of the condition. In 2006, one study compared [10] DM1 patients submitted to PTAs and patients kept under intensive insulin therapy, and showed that among non-proliferative retinopathy individuals, there was an even 50% ratio between improvement and stabilization, compared to 20% improvement, 10% stabilization and 70% worsening figures in the control group, under intensive insulin therapy. In turn, in PR patients or those submitted to laser surgery at a previous moment, stabilization rates were 86% of transplanted patients versus 43% in the control group. Contrarily, 14% of these patients presented worsening vs. 57% in the control group. However, other authors do not report a difference in retinopathy stabilization among patients with or without a functioning pancreatic graft, especially among patients with advanced retinopathy [11]. Our casuistry with 112 patients submitted to SPKT shows that improvement and stabilization occurred in 73.5%, primarily seen in non-proliferative retinopathy, with an important reduction in the number or ophthalmologic procedures after 04 years, except for the greater need of facectomy, perhaps resulting from the use of corticosteroids. (non-published data). Improvement-related factors are glycemic control, blood pressure control, lipid control and even a potential anti-inflammatory action of immunosuppressant.

## Summary: Pancreas Transplantationa and Diabetic Retinopathy

• May initially worsen.

• After four years or more, there is a reduction in the number of surgical procedures.

• Increase in the number of cases of cataract

• In the proliferative form, there is no consensus regarding benefits.

• Need for longer studies comprehending all forms of pancreas transplantations.

## Diabetic Neuropathy

Despite the high prevalence of diabetic neuropathy, not many papers involve an important number of patients. A classical study is Navarro et cols' [12], in which nearly 150 patients were followed for 10 years after transplantation, and a quick sensitive and motor improvement was observed, although the normalization of the parameters studied was not observed. Allen et cols. [13], when following up on 59 DM1 patients with SPKT for eight years, described similar outcomes to Navarro's.

Autonomic neuropathy, whose presence is associated to a higher mortality rate [14], may present an improvement which usually takes place after a prolonged period, nearly five years or more [12].

## Coronary and Peripheral Arterial Disease

Cardiovascular disease (CVD) is the main cause of death among patients with diabetes mellitus. Several studies show that glycemic control reduces the risk of microangiopathy in DM1 patients, but there are no prospective studies regarding CVD with a suitable patient population.

**Figure 1:**
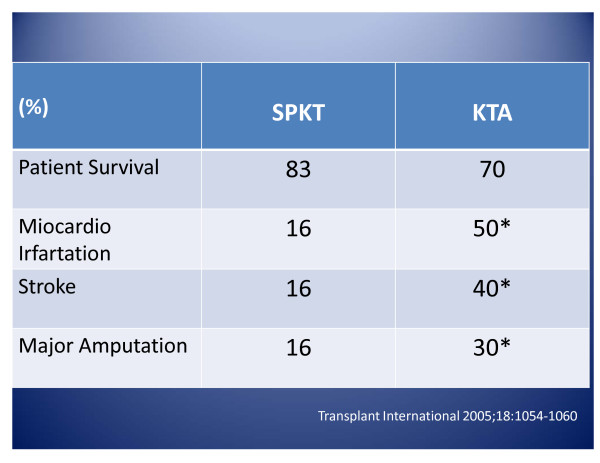
**CardioVascular Arterial Disease 10 years after SPKT compared to Kidney Transplantation Alone**:

CVD prevalence is very high in DM1 patients with renal disease, as observed by Oliveira et cols. [15] - this study evaluated patients in line for a SPKT, no prior history of angina and found 72% of patients with alterations in the coronary cardiac catheterism. Regarding patients participating in our program, in the pre-transplant phase, 9% performed angioplasty and 3%, open thoracic surgery. Following the SPKT, we noticed an important reduction in cardiovascular risk factors, represented by the metabolic syndrome defined by NCEP-III criteria, which, in our casuistry was only 11%. This finding can partly explain the reduction in mortality rates among these patients, if compared to DM patients submitted only to isolated kidney transplantations [16]. Fibrinogen is also reduced, as well as D-Dimers, homocysteine, triglycerides, von Willebrand factor in SPKT receptors, compared to isolated kidney receptors [17]. Other authors showed that in the five-year post operative period, the occurrence of vascular diseases is no different among SPKT and isolated kidney procedures, whereas after seven to ten years, patients submitted to SPKT present a significantly decreased incidence of acute myocardial infarction (2.4% to 16% vs. 17.5% to 50%), stroke (16% vs. 40%) and amputations (16% vs. 30%) [17-20]. Figure 1.

## Summary: Cardiovascular Disease and Pancreas Transplantation

• Most papers point to an improvement in CVD risk factors.

• Diastolic ventricular dysfunction improves after SPKT and PTA.

• CAD progression is milder in SPKT with a functioning pancreas.

• Mortality rates for SPKT are decreased when compared to patients submitted to isolated kidney transplantation from a deceased donor.

## Final considerations

Pancreas transplantation is an invasive procedure that can restore and maintain normoglycemic level very successfully and for a prolonged period in DM1 patients. The procedure elevates the morbimortality rates in the first few months following the surgery if compared to kidney transplants with living donors, but it offers a better quality of life to patients.

Although controversial, several studies have shown the stabilization or the improvement of some of the chronic complications related to diabetes, as well as the extra number of years of life that patients submitted to a double pancreas-kidney transplantation may gain.

Recent studies have demonstrated clashing outcomes regarding isolated pancreas transplantations, a fact which reinforces the need for a more discerning selection of patients for this procedure.

## Competing interests

The authors declare that they have no competing interests.

## Authors' contributions

JRS conceived the article, made indroduction, finished the article and adjusted the reviewers questions, PTM finished the article and adjusted the reviewers questions, EBR reviewed Diabetic Nephropathy, CSM reviewed Diabetic Retinopathy, AMG reviewed Diabetic Nephropathy, MML reviewed Diabetic Neuropathy, AS reviewed Diabetic Periferal Disease, MDFN reviewed Diabetic Coronary Disease, CS reviewed Diabetic Retinopathy, JOMP made final revision before sending to submittion. All authors read and approved the final manuscript.
